# Job demands and resources, work capabilities, and burnout of industrial psychology and human resource management practitioners

**DOI:** 10.3389/fpsyg.2026.1755805

**Published:** 2026-03-31

**Authors:** Sibusiso Leonard Mnxuma, Sebastiaan Rothmann, Marius Wilhelm Stander, Thapelo Chachaa

**Affiliations:** 1 Optentia Research Unit, North-West University, Vanderbijlpark, South Africa; 2 School of Industrial Psychology and Human Resource Management, North-West University, Vanderbijlpark, South Africa

**Keywords:** burnout, industrial psychologists, job demands, job resources, work capabilitiesjob demands, human resource management

## Abstract

Using a person-centered design, this study applies latent class analysis to identify distinct capability sets and examines their associations with job demands and resources, as well as burnout. A cross-sectional survey design was used with 203 industrial psychology and human resource management practitioners in South Africa. The Capability for Work Questionnaire, the Job Demands-Resources Scale, and the Burnout Assessment Tool were administered. Three capability sets were identified using latent class analysis: robust, constrained, and weak. Practitioners with robust and constrained capability sets reported significantly higher levels of co-worker support, role clarity, and autonomy compared to those with a weak capability set. Practitioners with a robust capability set experienced significantly greater career progress than those with constrained and weak capability sets. Importantly, those with a robust capability set (compared to the constrained and weak capability sets) reported significantly lower levels of exhaustion, mental distance, cognitive impairment, and emotional impairment. While individuals with a constrained capability set exhibited lower mental distance and emotional impairment than those with a weak capability set, the robust group consistently demonstrated the most favorable outcomes across all burnout indicators.

## 1 Introduction

Work contexts increasingly shape not only what employees do, but also what they are genuinely able to be and achieve through their work. From a capability perspective, individuals’ wellbeing and sustainable employability depend not merely on the presence of job resources, but on their real freedom to convert these conditions into valued work outcomes (Sen, 2001). The capability approach (CA) holds that what matters most, morally, is people’s freedom to achieve wellbeing and that wellbeing should be understood in terms of people’s functionings and capabilities (Robeyns and Byskov, 2025; Van der Klink et al., 2016).

There are three critical elements to the CA: capabilities, functionings, and agency (Robeyns, 2017; Sen, 2001). Capabilities refer to valued beings and doings that individuals are enabled for and that they can achieve (Nussbaum, 2011; Sen, 2001; Van der Klink et al., 2016). Functionings refer to the states and activities that comprise an individual’s being and doings. Agency refers to the possibility of shaping one’s life and living environment (process) and achieving valued opportunities (Sen, 2001). Beyond understanding what industrial psychology and human resource management (IRHRM) practitioners value, it is essential to determine whether they can achieve those outcomes (der Van Klink, 2019). When practitioners cannot attain what they regard as valuable from their work, they may develop burnout, which might threaten their sustainable employability (Gürbüz et al., 2023, 2024). Importantly, employees exposed to similar job demands and resources may nonetheless experience markedly different levels of wellbeing, depending on their capability sets, agency, and contextual conversion factors (Robeyns, 2017). This focus on heterogeneity is particularly relevant to IPHRM.

IPHRM practitioners are at the forefront of helping organizations navigate the complexities of the 21st century and aiding workforce sustainability, often while navigating high emotional, cognitive, and ethical demands themselves (Banerjee et al., 2023; Dhanpat and Stanz, 2024). These practitioners have to foster employee wellbeing, facilitate talent management, navigate hybrid work, and focus on telecommuting, digitization, productivity, and overall organizational effectiveness (Gupta, 2024; Scholze and Hecker, 2024). Yet their own capabilities to function sustainably at work are frequently assumed rather than examined (Cockcroft-McKay and Duchesne, 2021; Dattilio, 2015; Kristinsson et al., 2024; Skovholt and Trotter-Mathison, 2014). Understanding individual differences in how IPHRM practitioners are enabled or constrained to achieve valued work outcomes is, therefore, critical for advancing sustainable employability in this profession.

Although substantial research has examined job demands, resources, and burnout within the job demands-resources (JD-R) framework, less attention has been given to how available resources are converted into real opportunities for individuals to achieve valued work functionings (der Van Klink, 2019). The CA provides a useful lens for addressing this gap by focusing on the relationship between available resources and individuals’ freedom to realize meaningful work outcomes (Abma et al., 2016). In addition, most research in this area adopts variable-centered approaches that assume homogeneous relationships between work conditions and outcomes. Little research has employed person-centered approaches to explore heterogeneous configurations of work capabilities and how different practitioners experience their work contexts. The present study adopts a person-centered perspective (Portoghese et al., 2025), recognizing that IPHRM practitioners may experience different configurations of work capabilities (Ragadu and Rothmann, 2025). Accordingly, latent class analysis is used to identify distinct capability sets and to examine whether certain sets are associated with specific job demands and resources, as well as with a heightened risk of burnout.

The primary contribution of this study lies in the theoretical integration of the JD-R model with the CA. While the JD-R framework explains how job demands and resources relate to employee wellbeing, the CA provides a complementary perspective by focusing on how available resources are translated into real opportunities to achieve valued work outcomes. By combining these perspectives, the study examines how work capabilities function as a conceptual bridge between work conditions and employee functioning.

## 2 Literature overview

### 2.1 Work capabilities

The CA provides a human-centered framework that focuses on individuals’ freedom and real opportunities to achieve valued outcomes, not only on access to resources (Nussbaum, 2011; Sen, 2001). An enabling work environment allows individuals to achieve valued functionings (work capabilities), such as utilizing their skills and knowledge, contributing to meaningful goals, and establishing positive relationships (Abma et al., 2016; Van der Klink et al., 2016). These capabilities reflect both the structural aspects of the work environment and the individual’s ability to translate them into meaningful, valued outcomes.

Individuals’ capabilities refer to the combination of functionings that they are able and enabled to achieve (Murangi et al., 2022), given available resources and conversion factors (Van der Klink et al., 2016). As such, resources are helpful to the extent to which individuals are able and enabled to convert them into valuable work outcomes. In the absence of resource conversion, a misalignment arises between what IPHRM practitioners can do and what they are enabled to do. It is thus essential to note that, even in resource-rich environments, when IPHRM practitioners cannot convert these resources into tangible, valued functionings, the risk of burnout is heightened. As suggested by Van der Klink et al. (2016) and Abma et al. (2016), work capability consists of the following dimensions: (a) using knowledge and skills; (b) developing new knowledge and skills; (c) being involved in important decisions; (d) building and maintaining meaningful contacts at work; (e) setting own goals; (f) having a good income; and (g) contributing to something valuable.

According to der Van Klink (2019), the CA is an ethical framework central to social justice. Being well is not about what people have but about what they can do and be (Robeyns, 2017). Social justice entails combating injustices that prevent people from realizing what they value (Hocking, 2017). In a just society, people are treated equitably and have equal opportunities, not merely equal resources. Applying it to a work domain enables the identification of work-related values and the analysis of how individuals are enabled to achieve valued work outcomes. Even when individuals possess similar job resources, the extent to which they can convert them into valued functionings may differ significantly. Central to this are conversion factors such as individual characteristics (e.g., experience, confidence, and health), social dynamics (e.g., support from colleagues), and organizational structures (e.g., autonomy and role clarity). Understanding the interaction between the individual’s agency and the organizational context is essential.

#### 2.1.1 Job demands, job resources, and work capabilities of IPHRM practitioners

In this study, job demands and resources are treated as conditions that may help explain why practitioners belong to different work capability classes. This approach enables the identification of subgroups of IPHRM practitioners whose configurations of work capabilities are associated with varying levels of specific job demands and resources.

The JD-R model posits that every job has risk factors associated with wellbeing. These factors can be classified into two categories, namely, job demands and job resources (Bakker et al., 2005; Demerouti et al., 2001). Job demands refer to aspects of the job that require sustained effort, which might result in physiological, psychological, and emotional costs (Tummers and Bakker, 2021). Job resources are elements of the job that support the attainment of work goals, counteract job demands and associated costs, and promote personal learning, development, and growth (Demerouti and Bakker, 2023).

IPHRM practitioners play a crucial role in facilitating employee wellbeing, managing change, and driving strategic value within organizations (Huang, 2024; Whitsed et al., 2025). However, they must contend with various challenges inherent in their roles, including increased complexity and heightened expectations, which contribute to strategic outcomes and operational efficiency (Hoare and Vandenberghe, 2024), as well as the need to maintain professional composure and integrity (Gupta, 2024). Job demands common to IPHRM practitioners include high workloads, emotional labor, role conflict, and continuous professional development, arising from the rapidly changing world of work and shifts in workplace policies and labor laws (Anderson, 2022; McCormack et al., 2018).

The CA does not focus merely on resource availability, but rather on what individuals are able to do and be at work—that is, their capabilities and functionings (Gloss et al., 2017; Van der Klink et al., 2016). Few studies have integrated the JD-R model, the CA, and wellbeing (for exceptions see Barnard et al., 2023; Gürbüz et al., 2023; Murangi et al., 2022). Therefore, little is known about the associations between these constructs, particularly for IPHRM practitioners. Focusing on capabilities and functionings does not disregard the importance of resources, particularly as emphasized by the JD-R model. Resources are crucial to the CA, as capabilities depend on resources.

Resources should be assessed for their effectiveness in enhancing the lives and liberties of people rather than being valued solely for their intrinsic worth (Barnard et al., 2023). From this perspective, work capabilities can be understood as an intermediate outcome of the job demands–resources dynamic, reflecting how working conditions shape what individuals are effectively able to be and do at work. Specific job resources, such as autonomy (Duong and Pham, 2023), role clarity (De Villiers and Stander, 2011), and support from colleagues, may enhance individuals’ ability to realize valued beings and doings, including using and developing their knowledge and skills, participating in important decisions, setting and pursuing personal goals, building and maintaining meaningful relationships, earning a sufficient income, and contributing to work that is experienced as valuable (Abma et al., 2016; Van der Klink et al., 2016; der Van Klink, 2019).

#### 2.1.2 Burnout

Twenty-first century workplaces are characterized by high volatility, uncertainty, complexity, and ambiguity (VUCA), presenting unique challenges to IPHRM practitioners (Ghislieri et al., 2018; Volderauer et al., 2024). The dynamic nature of VUCA workplaces results in specific job demands influencing the ability of IPHRM practitioners to perform their roles while maintaining their wellbeing (McCormack et al., 2018; Scholze and Hecker, 2024). Burnout has emerged as a significant concern in this context.

The risk of burnout in helping and people-oriented professions is well-documented (Barnard et al., 2023; Kaiser et al., 2020; Rupert and Kent, 2007), and this concern is now extending to IPHRM practitioners as well (Kristinsson et al., 2024; McCormack et al., 2018). Burnout is a state of work-related exhaustion among employees, characterized by tiredness, reduced ability to self-regulate emotional and cognitive processes, and mental distancing (De Beer et al., 2022). Studies show growing interest in burnout, given its effects on outcomes such as high turnover, lower-quality care for employees, and ethical risks associated with poor decision-making (Anderson, 2022; Barnard et al., 2023; Murangi et al., 2022; Rogozińska-Pawełczyk, 2024). Research has shown that burnout is closely related to the intention to leave work (Gonzales et al., 2025), which affects the sustainable employability of IPHRM practitioners (Bakker et al., 2014; Gürbüz et al., 2024; Van der Klink et al., 2016). Although no recent studies on burnout among IPHRM practitioners in South Africa were found, van Zyl et al. (2020) reported that industrial psychologists exhibited distinct mental health profiles. These profiles co-varied with meaningful work and work engagement—factors closely related to burnout. In this regard, Ferrer et al. (2024) argue that human resource work has intensified globally with “extreme work” that raises job demands and resource risks: high workload, emotional demands, and role conflict.

It is essential to investigate the effects of systemic factors (Ungar, 2024), such as workload, role clarity, job autonomy, and co-worker relationships, on the burnout and sustainable employability of IPHRM practitioners (Demerouti and Bakker, 2023; Gürbüz et al., 2024; Kristinsson et al., 2024; Nilsson and Nilsson, 2021; Tummers and Bakker, 2021). The JD-R model offers a robust and widely used framework (Bakker et al., 2023; Barnard et al., 2023; McCormack et al., 2018) for identifying job characteristics (demands and resources) that affect work capabilities and burnout (Demerouti and Bakker, 2023). Excessive demands can lead to burnout (Barnard et al., 2023), while a lack of job resources makes it difficult for individuals to meet demands, thereby increasing the risk of burnout. However, the JD-R model does not thoroughly account for the effects of context and individual agency. The model assumes homogeneity and does not account for individual differences in how practitioners with similar demands and resources may respond differently (Robeyns, 2017; Van Veldhoven et al., 2020). It assumes that high job demands and limited resources lead to burnout, and that sufficient resources will eliminate the risk of burnout (Ji et al., 2024; Li et al., 2025). This assumption of a linear causal relationship among job demands, resources, and burnout does not account for the conversion of these resources into meaningful and valuable work outcomes. Therefore, existing burnout models offer limited insight into the mechanisms through which job demands and resources translate into wellbeing outcomes.

Bradshaw and Ryan (2026) explain that when individuals feel controlled, it undermines their sense of autonomy, which results in feelings of alienation and burnout. Autonomy is not about the absence of external influences, but about the ability to integrate them meaningfully into one’s values (Kuzborska et al., 2025). Becoming aware of one’s values and acting with a sense of willingness are requirements of such integration. Providing individuals with specific resources, capabilities, and rights that allow them to pursue what matters to them can support autonomy and justice (Nussbaum, 2011; Sen, 2005). Martela and Ryan (2024) showed that greater autonomy and need satisfaction uniformly and robustly predicted wellbeing across countries. Building on these perspectives, the JD-R model has emerged as a widely adopted framework.

#### 2.1.3 Current study

Although job resources and capabilities are related constructs, they capture different aspects of the work experience (der Van Klink, 2019). Job resources refer to features of the work environment that support goal attainment or personal development, such as autonomy, role clarity, and social support. Capabilities, by contrast, refer to individuals’ perceived opportunities to realize valued work functionings (Abma et al., 2016). In this sense, capabilities capture the extent to which available work conditions can be translated into meaningful opportunities for action. The capability perspective, therefore, complements existing mediators frequently examined in organizational research. Whereas constructs such as engagement or meaningful work describe psychological states or outcomes of work experiences, capabilities capture the opportunity structure that precedes these outcomes (Van der Klink et al., 2016). By focusing on individuals’ perceived freedom to achieve valued work outcomes, the CA helps explain why similar work conditions may lead to different wellbeing outcomes across individuals. In this way, capabilities offer a useful bridge between work conditions and employee functioning.

No studies have integrated the JD-R model with the CA to examine burnout and sustainable employability among IPHRM practitioners. This study adopted a person-centered approach to examine individual differences in work capability configurations among IPHRM practitioners. Using the CA as its primary theoretical lens, the study identified distinct capability sets and examined how these sets differed in terms of job demands, job resources, and burnout experiences.

Van der Klink et al. (2016) posit that sustainable employability depends on whether resources are enabled, valued, and achieved rather than merely being present. IPHRM practitioners who lack specific capabilities (e.g., the inability to influence decisions, a lack of purpose, and limited opportunities for creativity) are at risk of experiencing burnout, not just those with high job demands. Integrating the CA and the JD-R model allows for a more nuanced understanding. The opposite can also be true: when job demands are high, and resources are scarce or unusable, the capability to function effectively may be hindered, resulting in an increased risk of burnout (Abma et al., 2016). Barnard et al. (2023) found that specific resources, including job autonomy, supervisor and co-worker relationships, and access to learning opportunities, were significantly associated with capabilities, such as using and developing knowledge, involvement in decision-making, and building meaningful relationships at work. Traditional interventions focus on reducing job demands and/or increasing generic resources (Bakker et al., 2023). Although these strategies have merit, their impact is limited and short-term, particularly in the dynamic and high-stakes environment where IPHRM practitioners work. Resources become meaningful when they are valued, enabled, and achieved, contributing to a capability set (der Van Klink, 2019). When resources are not aligned with what individuals value in their professional lives, they are likely to remain unused or ineffective in preventing adverse outcomes, such as burnout. For interventions to support the sustainable employability of IPHRM practitioners and prevent burnout in a more enduring way, a shift should be made from the mere reduction of demands or the increase in generic resources to designing work environments that enable capabilities (job crafting, freedom to pursue professional goals, and involvement in decision-making) (Murangi et al., 2022).

This study contributes to scientific knowledge by integrating job demands and resources with the work capabilities and burnout of IPHRM practitioners. Utilizing a person-centered approach, specifically latent class analysis (LCA), this study moves beyond the traditional variable-centered methodologies to discover distinct capability sets of IPHRM practitioners (Barnard et al., 2023; Caesens et al., 2020). To avoid the assumption of a “one-size-fits-all” resource utilization model, a person-centered approach to work capabilities enables a nuanced examination of how different subgroups of practitioners’ experience, value, and convert job resources into capability sets, which can then be studied in relation to job demands and resources as well as burnout.

The following hypotheses were set:

Hypothesis 1: job demands are associated with IPHRM practitioners’ capability sets.

Hypothesis 2: job resources are associated with IPHRM practitioners’ capability sets.

Hypothesis 3: work capability sets of IPHRM practitioners are associated with exhaustion (Hypothesis 3a), mental distance (Hypothesis 3b), cognitive impairment (Hypothesis 3c), and emotional impairment (Hypothesis 3d).

## 3 Materials and methods

### 3.1 Participants

A sample of IPHRM practitioners based in South Africa participated in the study. The total sample size obtained was 205. The demographic characteristics of the IPHRM practitioners are described in Table 1.

**Table 1:** Description of the participants.

Variable	Grouping	Observations (*n*)	Frequency (%)
Gender	Male Female Other	64 139 2	31.2 67.8 1
Age group	20–30 years 31–40 years 41–50 years 51 and older Missing values	32 91 53 26 3	15.6 44.4 25.9 12.7 1.5
Language	Afrikaans English African languages	64 57 84	31.5 27.8 40.9
Years of experience	Less than 2 years 2–5 years More than 5 years Missing values	50 66 72 1	24.4 32.2 42.9 0.5

Most of the participants were female (67.8%). The participants’ ages ranged from 20 to 67 years, with the majority aged 31–40 years (44.4%). African home-language-speaking participants (40.9%) comprised the largest part of the sample, followed by the Afrikaans language group (31.5%). Regarding qualification, most participants had been qualified for 11 years or more (42.9%), followed by those qualified for six to 10 years (23.6%). Concerning work experience, 35.6% of the participants had six to 10 years of experience, while 32.7% had two to 5 years of work experience.

### 3.2 Measuring instruments

A biographical questionnaire was used to gather demographic data. This included information on participants’ gender, age, first language, highest level of education, employment contract, years of experience in IPHRM, and the sector in which they operated.

The Job Demands-Resources Scale (JDRS) (Rothmann et al., 2006) was used to assess the participants’ job demands and resources. A selection of three items per dimension, representing demands and resources applicable to the work of IPHRM practitioners, was taken from the questionnaire. The demands included (a) quantitative demands (e.g., “Do you have too much work?”), (b) cognitive demands (e.g., “Do you have to remember many things in your work?”), and (c) emotional demands (e.g., “Are you confronted in your work with things that affect you personally?”). The job resources included (a) autonomy (e.g., “Does your job offer you the possibility of independent thought and action?”), (b) relationships with colleagues (e.g., “If necessary, can you ask your colleagues for help?”), (c) role clarity (e.g., “Do you know exactly for what you are responsible?”), and (d) career progress (e.g., “Does your work give you the opportunity to be promoted?”). Participants responded to the items using a five-point Likert scale, ranging from 1 (*never*) to 5 (*always*). The JDRS is valid, reliable, and invariant for organizations in South Africa (Rothmann et al., 2006). The alpha coefficients of the scales vary from 0.78 to 0.89 (Barnard et al., 2023).

The Capability Set for Work Questionnaire (CSWQ) (Abma et al., 2016) was administered to evaluate the work capabilities and capability sets of IPHRM practitioners. It consists of 21 items assessing seven capability aspects: (a) using knowledge and skills; (b) developing new knowledge and skills; (c) being involved in important decisions; (d) building and maintaining meaningful contacts at work; (e) setting own goals; (f) having a good income; and (g) contributing to something valuable. Each of the seven capability aspects was measured using three similar statements: (a) whether participants considered the aspect important (reflecting its perceived value), (b) whether their work provided sufficient opportunities to pursue it (indicating an enabling work environment), and (c) whether they were able to achieve it (reflecting their capacity and competence to realize it). On a five-point Likert scale, respondents indicated the extent to which they agreed, ranging from 1 (*totally not*) to 5 (*to a great extent*).

In line with the recommendations of Abma et al. (2016) a capability aspect (rated from 1 to 5) was counted as part of the capability set when three conditions were met: participants saw it as important (A = 4–5), the workplace offered enough opportunities to pursue it (B = 4–5), and they were able to realize it in practice (C = 4–5). A capability aspect was not included in the capability set when any of the following applied: (a) it was important to the person (A = 4–5) but the workplace did not provide enough opportunities (B ≤ 3); (b) it was important (A = 4–5) but the person was unable to achieve it (C ≤ 3); (c) the workplace offered opportunities (B = 4–5) but the person could not make use of them (C ≤ 3). The CSWQ is a valid instrument for measuring IPHRM practitioners’ capability set and determining correlations with other constructs, such as work role functioning-flexibility demands (−0.19), workability (−0.30), and work performance (−0.28) (Abma et al., 2016).

The 12-item version of the Burnout Assessment Tool (BAT12) (Hadžibajramović et al., 2020; Schaufeli et al., 2020) was used to measure participants’ burnout. The BAT12 assessed four dimensions: exhaustion (e.g., “At work, I felt mentally exhausted”), mental distance (e.g., “I struggled to find any enthusiasm for my work”), cognitive impairment (e.g., “At work, I had trouble staying focused”), and emotional impairment (e.g., “At work, I felt unable to control my emotions”). Participants rated the items using a Likert scale, ranging from 1 (*never*) to 5 (*always*). Hadžibajramović et al. (2020) reported that the BAT12 demonstrated acceptable psychometric properties. Schaufeli et al. (2020) found the following reliability coefficients for the subscales: exhaustion (*α* = 0.92), mental distance (*α* = 0.91), cognitive impairment (*α* = 0.92), and emotional impairment (*α* = 0.90). De Beer et al. (2022) concluded that the BAT12 was a valid and reliable burnout measure.

### 3.3 Research procedure

The North-West University Education, Management and Economic Sciences, Law, Theology, Engineering and Natural Sciences Research Ethics Committee (NWU-EMELTEN-REC) granted ethics clearance for the study (NWU-00418-23-A2). The researcher approached two professional bodies, namely, the Society for Industrial/Organizational Psychology of South Africa and the South African Board of People Practice, for permission to conduct the research. On approval, the researchers provided the professional bodies with an invitation to participate in the study and share it with their members via various media, including email, newsletters, and LinkedIn. The purpose, data collection procedure, and ethical and legal considerations (including confidentiality, anonymity, and the voluntary nature of participation) of the study were communicated to potential participants.

The survey was administered through an online platform. A link to the informed consent form was included; participants were granted access to the questionnaire only after completing the informed consent form section. The survey responses were downloaded to the researcher’s computer in Excel format and saved in a password-protected folder. Data will be stored for 5 years, after which it will be destroyed.

### 3.4 Statistical analyses

Data were analyzed using Mplus 9.0 (Muthén and Muthén, 1998–2025) and SPSS 30 (IBM Corp., 2020). Confirmatory factor analysis (CFA) was used to investigate the factor structures of the measures. The weighted least squares means and variance adjusted estimator (WLSMV), which is suitable for the analysis of ordinal data was used. Indices that were used to assess the model fit include: the chi-square statistic (a measure of absolute model fit), the Tucker-Lewis index (TLI), the comparative fit index (CFI), the standardized root mean residual (SRMR), and the root mean square error of approximation (RMSEA) (West et al., 2023). The TLI and CFI values must be greater than 0.90; scores greater than 0.95 indicate excellent fit. Moreover, RMSEA and SRMR values less than 0.08 indicate a good fit between the model and the data (West et al., 2023).

Latent class analysis was employed in Mplus 9.0 (Muthén and Muthén, 1998–2025). The use of latent class analysis is consistent with the CA’s emphasis on heterogeneity in individuals’ opportunities to achieve valued functionings. Rather than assuming that work conditions affect all individuals in the same way, the capability model recognizes that people experience different configurations of opportunities depending on available resources, personal circumstances, and conversion factors. Therefore, a person-centered analytical approach provides a useful way to identify distinct capability sets within the population. By grouping individuals according to patterns in their capability sets, latent class analysis captures variation in the opportunity structures through which individuals exercise agency at work (Spurk et al., 2020).

Several models with increasing latent classes were tested. A model was retained when it significantly improved on the reference model. Models were compared using the Akaike information criterion (AIC) and Bayesian information criterion (BIC), adjusted Bayesian information criterion (ABIC) values, the Lo–Mendell–Rubin (LMR) LR test (Lo et al., 2001), the adjusted LMR LR test (ALMR), and the bootstrapped likelihood ratio test (BLRT) (Wang and Wang, 2020). The AIC, BIC, and ABIC with the lowest values indicate the best-fitting model. Entropy values (ranging from 0 to 1) were used to assess how clearly each model separates people into classes; a value of 1 indicates a good classification, while a value lower than 0.60 is unacceptable (Geiser, 2010).

Descriptive statistics (frequencies, means, and standard deviations) and Pearson correlation coefficients were computed using SPSS 30 (IBM Corp., 2020). Scale reliability estimates were computed, and a cut-off of 0.70 was used (Nunnally and Bernstein, 1994). Cross-tabulations were used to assess the associations between capability sets (latent classes) and selected biographical variables (Field, 2024). Using the automatic Block, Croon, and Hagenaars (BCH) method, the means of the distal continuous outcome were compared across latent classes (Asparouhov and Muthén, 2014; Bakk and Vermunt, 2016).

## 4 Results

### 4.1 Capabilities of participants

Table 2 reports the frequencies and percentages of participants’ capabilities.

**Table 2:** Capabilities of participants.

Capability	Code	Importance	Opportunity	Achievement	Capability	Combined
Use of knowledge and skills	0 1	9.4 90.6	23.2 76.8	23.6 76.4	34.0 66.0	Not capable Capable
Development of new knowledge and skills	0 1	8.4 91.6	26.1 73.9	28.6 71.4	35.0 65.0	Not capable Capable
Involvement in important decisions	0 1	13.8 86.2	36.0 64.0	40.9 59.1	46.3 53.7	Not capable Capable
Meaningful contacts at work	0 1	13.3 86.7	26.6 73.4	23.6 76.4	36.5 63.5	Not capable Capable
Setting own goals	0 1	11.8 88.2	22.2 77.8	25.1 74.9	36.5 63.5	Not capable Capable
Earning a good income	0 1	15.3 84.7	40.9 59.1	48.3 51.7	55.2 44.8	Not capable Capable
Contributing to something valuable	0 1	10.8 89.2	29.1 70.9	31.0 69.0	36.5 63.5	Not capable Capable

Code = 0 if the participant’s score was ≤3; Code = 1 if the participant’s score ≥4.

Table 2 indicates that the participants considered all capabilities as important. The results ranged between 84.7% for earning a good income and 91.6% for developing new knowledge and skills. The results for enabling values (opportunity) varied slightly, ranging between 59.1% for earning a good income and 77.8% for setting own goals. The results regarding the level of achievement ranged from 51.7% (having a good income) to 76.4% (utilizing knowledge and skills), reflecting a significant gap between the value placed on specific capabilities and the extent to which they were achieved. The highest capabilities of participants included using knowledge and skills (66%), developing new knowledge and skills (65%), developing and maintaining meaningful work contacts (63.5%), setting personal goals (63.5%), and contributing to something valuable (63.5%). Table 2 shows that the lowest reported capabilities among participants were earning a good income (44.8%) and involvement in important decisions (53.7%), patterns that may reflect broader structural or systemic conditions related to autonomy and financial wellbeing.

### 4.2 Confirmatory factor analysis

The factor structure of each instrument was first evaluated separately to establish that each scale demonstrated the intended dimensionality in the sample and to identify item-level sources of misfit in a controlled manner (Brown, 2015). Subsequently, a full measurement model including all constructs was estimated simultaneously to evaluate overall measurement fit and, importantly, discriminant validity among the latent variables, before proceeding to the structural model (Kline, 2023).

#### 4.2.1 Evaluation of the separate measurement models

CFA was employed to test the measurement model of the JDRS and BAT. The measurement models for the JDRS were specified as follows: (a) Model 1 consisted of six latent variables, representing two job demands and four job resources. The two job demands were labelled cognitive demands (measured by five items) and emotional demands (measured by three items). The four job resources were labelled relationship with colleagues (measured by three items), role clarity (measured by three items), career progress (measured by three items), and autonomy (measured by three items). (b) Model 2 consisted of two latent variables, representing job demands (eight items) and job resources (12 items).

The measurement models for the BAT were specified as follows: (a) Model 1 consisted of four latent variables, namely, exhaustion (measured by eight items), mental distance (measured by five items), cognitive impairment (measured by five items), and emotional impairment (measured by five items). (b) Model 2 consisted of one latent variable, representing burnout, which was measured by 23 items.

Table 3 shows the results of the confirmatory factor analyses of the JDRS and the BAT. Concerning the JDRS, Model 1 showed the best fit. The fit statistics of Model 1 were as follows: *χ*^2^ = 323.50, *df* = 155, *p* > 0.001; CFI = 0.95; TLI = 0.94; RMSEA = 0.07, *p* < 0.001 [0.06, 0.08]; and SRMR = 0.07. Regarding the BAT, Model 1 showed the best fit. The fit statistics of Model 1 were as follows: *χ*^2^ = 474.68, *df* = 224, *p* < 0.001; CFI = 0.96; TLI = 0.95; RMSEA = 0.07, *p* < 0.001 [0.07, 0.08]; and SRMR = 0.06.

**Table 3:** CFA of the JDRS and the BAT.

Model	*χ* ^2^	*df*	CFI	TLI	RMSEA	SRMR
JDRS						
1	323.50	155	0.95	0.94	0.07 [0.06, 0.08]	0.07
2	830.30	169	0.81	0.79	0.14* [0.13, 0.15]	0.12
BAT						
1	474.68	224	0.96	0.95	0.07 [0.07, 0.08]	0.06
2	988.99	230	0.87	0.86	0.13* [0.12, 0.14]	0.09

*p < 0.01.

Table 4 shows the standardized beta coefficients of the variables.

**Table 4:** Standardized regression coefficients of the JDRS and BAT.

Variable	*β*	Variable	*β*	Variable	*β*	Variable	*β*
Cognitive demands		Career progress		Exhaustion		Cognitive impairment	
JDR1	0.71*	JDR22	0.68*	BAT1	0.69*	BAT14	0.76*
JDR2	0.81*	JDR23	0.71*	BAT2	0.19*	BAT15	0.87*
JDR3	0.86*	JDR24	0.66*	BAT3	0.70*	BAT16	0.76*
JDR11	0.63*	Autonomy		BAT4	0.75*	BAT17	0.79*
JDR12	0.56*	JDR25	0.86*	BAT5	0.82*	BAT18	0.71*
Emotional demands		JDR26	0.92*	BAT6	0.81*	Emotional impairment	
JDR7	0.55*	JDR28	0.80*	BAT7	0.79*	BAT19	0.74*
JDR8	0.69*			BAT8	0.76*	BAT20	0.81*
JDR9	0.83*			Mental distance		BAT21	0.66*
Role clarity				BAT9	0.78*	BAT22	0.83*
JDR19	0.75*			BAT10	0.69*	BAT23	0.71*
JDR20	0.85*			BAT11	0.80*		
JDR21	0.87*			BAT12	0.86*		
Relationship: colleagues				BAT13	0.67*		
JDR13	0.83*						
JDR14	0.88*						
JDR15	0.74*						

*p < 0.001.

Table 4 shows that the items of the three scales loaded statistically significantly on the latent variables on which they were supposed to load. All items, except BAT2, showed standardized estimates >0.50.

#### 4.2.2 Evaluation of the full measurement model

Subsequently, a full measurement model was estimated to assess overall measurement fit, comprising six latent variables representing job demands and job resources and four latent variables representing burnout. The indicator variables for all latent variables were identical to those used in the separate measurement models. The following fit statistics were obtained: *χ*^2^(815) = 1,345.33, *p* < 0.001; RMSEA = 0.06, 90% CI [0.05, 0.06], *p* = 0.025; SRMR = 0.07.

### 4.3 Descriptive statistics, reliabilities, and correlations of the scales

Table 5 shows the descriptive statistics, reliabilities, and Pearson correlations of the variables. The reliability coefficients for the three scales used in this study were acceptable, with values greater than 0.70 (Nunnally and Bernstein, 1994).

**Table 5:** Descriptive statistics, reliabilities, and correlations of the scales (*N* = 205).

Variable	*ω*	Mean	SD	1	2	3	4	5	6	7	8	9	10
1. Cognitive demands	0.79	3.75	0.77	–	–	–	–	–	–	–	–	–	–
2. Emotional demands	0.70	3.04	0.82	0.43**	–	–	–	–	–	–	–	–	–
3. Relation: colleagues	0.82	3.75	0.92	0.07	−0.21**	–	–	–	–	–	–	–	–
4. Role clarity	0.81	3.99	0.88	0.17*	−0.15*	0.35**	–	–	–	–	–	–	–
5. Career progress	0.68	3.01	1.01	0.13	−0.03	0.21**	0.23**	–	–	–	–	–	–
6. Autonomy	0.86	3.80	0.98	0.17*	−0.17*	0.46**	0.49**	0.42**	–	–	–	–	–
7. Capability set	0.88	0.60	0.37	0.25*	−0.09	0.43**	0.46**	0.35**	0.55**	–	–	–	–
8. Exhaustion	0.87	2.96	0.77	0.29**	0.54**	−0.36**	−0.29**	−0.24**	−0.35**	−0.29**	–	–	–
9. Mental distance	0.84	2.43	0.90	−0.01	0.36**	−0.41**	−0.44**	−0.28**	−0.48**	−0.53**	0.62**	–	–
10. Cognitive impairment	0.84	2.42	0.82	−0.03	0.34**	−0.33**	−0.43**	−0.19**	−0.36**	−0.37**	0.59**	0.59**	–
11. Emotional impairment	0.82	2.07	0.81	−0.17*	0.30**	−0.39**	−0.47**	−0.17*	−0.42**	−0.48**	0.40**	0.63**	0.60**

**p < 0.01; *p < 0.05; *r* < 0.30 = small effect; 0.30 < *r* < 0.50 = medium effect; *r* > 0.50 = large effect.

Cognitive demands were positively associated with emotional demands (medium effect). Emotional demands were positively associated with exhaustion (large effect) and mental distance, cognitive impairment, and emotional impairment (all medium effects). The capability set was statistically significantly and positively associated with autonomy (large effect) and relations with colleagues, role clarity, and career progress (all medium effects), and negatively associated with mental distance (large effect) and cognitive and emotional impairment (medium effects). Relations with colleagues, role clarity, and autonomy were statistically significantly and negatively associated with mental distance and cognitive and emotional impairment (all medium effects). Lastly, the capability set was statistically significantly and negatively associated with mental distance (large effect) and with cognitive and emotional impairment (medium effects).

### 4.4 Latent class analysis

Latent class analysis (LCA) was conducted in Mplus 9.0 using the seven capabilities. Models with increasing numbers of latent classes were tested. (see Table 6.)

**Table 6:** Comparison of capability classes.

Class	AIC	BIC	ABIC	LMR LR test *p*-value	ALMR LR test *p*-value	BLRT *p*-value	Entropy	Smallest class membership (%)
1-Class	1,912.85	1,936.11	1,913.93	–	–	–	–	–
2-Class	1,434.20	1,484.04	1,436.52	0.000**	0.000**	0.000	0.93	35.44
3-Class	1,417.08	1,493.51	1,420.63	0.061	0.065	0.000	0.80	21.36
4-Class	1,410.39	1,513.40	1,415.18	0.111	0.116	0.013	0.86	2.43
5-Class	1,412.12	1,541.72	1,418.16	0.573	0.577	0.333	0.91	2.43

AIC = Akaike information criterion; BIC = Bayesian information criterion; ABIC = adjusted Bayesian information criterion; LMR LR = Lo–Mendell–Rubin test; ALMR LR = adjusted Lo–Mendell–Rubin test; BLRT = bootstrapped likelihood ratio test; **p < 0.01.

**Figure 1:**
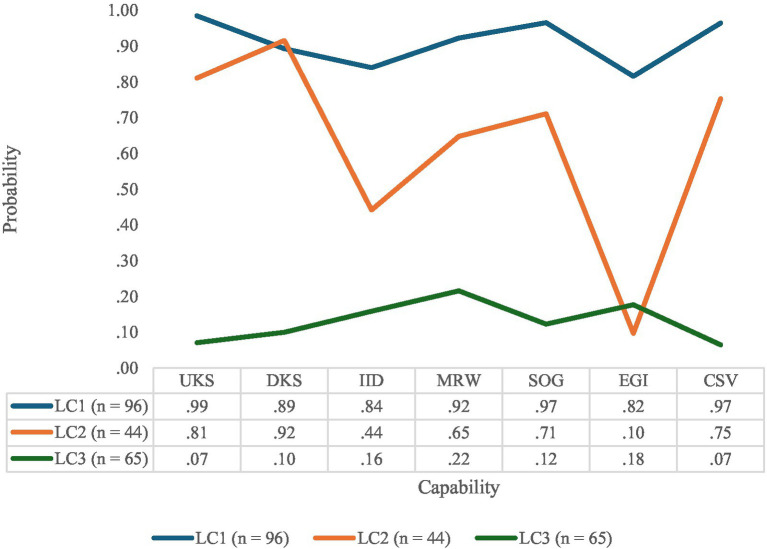
Latent classes of capability sets of IPHRM practitioners. UKS = using knowledge and skills; DKS = developing new knowledge and skills; IID = being involved in important decisions; MRW = building and maintaining meaningful relationships at work; SOG = setting own goals; EGI = earning a good income; CSV = contributing to something valuable.

From Table 6, it is evident that the two-class solution had a better fit than the one-class solution (ΔAIC = −478.65; ΔBIC = −452.07; and ΔABIC = −477.41). Additionally, the LMR, ALMR, and BLRT of the two-class model were statistically significant (*p* < 0.01). This indicates that the two-class model fitted the data better than the one-class model. The model with three classes was significantly better than the two-class solution (ΔAIC = −17.12; ΔBIC = −9.47; ΔABIC = 15.89). However, while the *p*-value for BLRT (*p* < 0.001) remained statistically significant, the *p*-values for the LMR (*p* = 0.061) and ALMR (*p* = 0.065) were not. Furthermore, the four-class model provided a better fit compared to the three-class solution when the contrast was based on AIC and ABIC values (ΔAIC = −6.69; ΔABIC = −5.45). However, the smallest class membership did not justify a four-class model. Lastly, the model with five classes was not better than the four-class model (ΔAIC = 1.73; ΔBIC = 28.32; ΔABIC = 2.98), with all *p*-values statistically non-significant (*p* > 0.05). The class proportions were acceptable for Classes 1, 2, and 3. The entropy value of 0.86 for three classes indicated an acceptable classification (Wang and Wang, 2020). The average latent class probabilities were also acceptable: Class 1 = 0.92; Class 2 = 0.83; and Class 3 = 0.97. The three classes are illustrated in Figure 1.

The three classes in Figure 1 are as follows: Latent Class 1 (robust capability set; 46.82%) showed a high probability of endorsing all capabilities. Latent Class 2 (constrained capability set; 21.46%) showed a high probability of endorsing two capabilities (namely, the use of knowledge and skills and developing new knowledge and skills), a low probability of endorsing the capability of earning a good income, and a moderate probability of endorsing the remaining capabilities. Latent Class 3 (the weak capability set) represented 31.71% of the participants. This group showed a low probability of endorsing all capabilities.

Cross-tabulations were used to examine the associations between capability sets, as identified through latent class analysis, and two biographical variables, namely gender and work experience. These variables were selected because cell sizes for other biographical characteristics were too small to permit reliable analysis. Gender was categorized as male or female, and work experience was grouped into three categories: less than 2 years, 2–5 years, and 6 years or more. Both variables were considered theoretically relevant, as differences in gender and professional experience may be associated with access to resources and opportunities that shape IPHRM practitioners’ work capabilities. Chi-square tests of independence indicated no statistically significant association between gender and capability sets, *χ*^2^(2) = 0.51, *p* = 0.777. Similarly, no statistically significant association was found between work experience and capability sets, *χ*^2^(4) = 6.70, *p* = 0.153. These results suggest that, within the present sample, membership in capability sets was not systematically associated with practitioners’ gender or level of work experience.

### 4.5 Latent classes and distal outcomes

This study calculated the mean of a continuous distal outcome across multiple latent classes by applying the automatic BCH approach (Asparouhov and Muthén, 2014; Bakk and Vermunt, 2016). Table 7 shows the equality tests of means of the different variables across latent classes.

**Table 7:** Equality tests of means across latent classes of IPHRM practitioners.

Cognitive demands	Mean	SE	Emotional demands	Mean	SE
Robust (1)	3.92	0.08	Robust (1)	2.97	0.09
Constrained (2)	3.80	0.14	Constrained (2)	3.09	0.16
Weak (3)	3.46	0.10	Weak (3)	3.12	0.10
Overall test: *χ*^2^ = 13.32^**^, comparison of classes: 1 > 3^**^			Overall test: *χ*^2^ = 1.11, comparison of classes: NS		
Relationships with colleagues	Mean	SE	Role clarity	Mean	SE
Robust (1)	4.03	0.09	Robust (1)	4.28	0.08
Constrained (2)	3.98	0.14	Constrained (2)	4.20	0.15
Weak (3)	3.16	0.12	Weak (3)	3.40	0.13
Overall test: *χ*^2^ = 36.11^**^, comparison of classes: 1 > 3^**^, 2 > 3^**^			Overall test: *χ*^2^ = 37.94^**^, comparison of classes: 1 > 3^**^, 2 > 3^**^		
Career progress	Mean	SE	Autonomy	Mean	SE
Robust (1)	3.55	0.11	Robust (1)	4.23	0.08
Constrained (2)	2.48	0.17	Constrained (2)	4.05	0.15
Weak (3)	2.59	0.11	Weak (3)	2.96	0.13
Overall test: *χ*^2^ = 43.98^**^, comparison of classes: 1 > 2^**^, 1 > 3^**^			Overall test: *χ*^2^ = 72.91^**^, comparison of classes: 1 > 3^**^, 2 > 3^**^		
Exhaustion	Mean	SE	Mental distance	Mean	SE
Robust (1)	2.71	0.15	Robust (1)	1.97	0.09
Constrained (2)	3.16	0.09	Constrained (2)	2.45	0.14
Weak (3)	3.19	0.08	Weak (3)	3.11	0.10
Overall test: *χ*^2^ = 15.96^**^, comparison of classes: 1 < 3^**^			Overall test: *χ*^2^ = 75.03^**^, comparison of classes: 1 < 2^*^, 1 < 3^**^, 2 < 3^**^		
Cognitive impairment	Mean	SE	Emotional impairment	Mean	SE
Robust	2.12	0.09	Robust	1.78	0.08
Constrained	2.58	0.14	Constrained	1.90	0.13
Weak	2.76	0.11	Weak	2.63	0.10
Overall test: *χ*^2^ = 23.32^**^, comparison of classes: 1 < 2^*^, 1 < 3^**^			Overall test: *χ*^2^ = 49.39^**^, comparison of classes: 1 < 3^**^, 2 < 3^**^		

*p < 0.01; **p < 0.001, NS = non-significant.

Regarding job demands and resources, Table 7 shows that the latent classes differed significantly in terms of cognitive demands, relationships with colleagues, role clarity, career progress, and autonomy. The robust capability set, compared to the weak capability set, experienced statistically significantly higher cognitive demands (*χ*^2^ = 13.01, *p* < 0.001). Concerning job resources, IPHRMs with a robust capability set and those with a constrained capability set experienced statistically significantly higher levels of the following job resources compared to the weak capability set: co-worker support (*χ*^2^_Robust_ = 32.22, *p* < 0.001; *χ*^2^_Constrained_ = 19.05, *p* < 0.001), role clarity (*χ*^2^_Robust_ = 36.60, *p* < 0.001; *χ*^2^_Constrained_ = 15.79, *p* < 0.001), and autonomy (*χ*^2^_Robust_ = 71.55, *p* < 0.001; *χ*^2^_Constrained_ = 20.04, *p* < 0.001). Furthermore, the robust capability set experienced statistically significantly higher levels of career progress compared to the constrained (*χ*^2^ = 23.21, *p* < 0.001) and weak (*χ*^2^ = 38.21, *p* < 0.001) capability sets.

Regarding burnout, IPHRMs with a robust capability set experienced statistically significantly lower levels of the following variables compared to the constrained and weak capability sets: exhaustion (*χ*^2^_Constrained_ = 5.71, *p* < 0.001; *χ*^2^_Weak_ = 15.31, *p* < 0.011), mental distance (*χ*^2^_Constrained_ = 6.83, *p* < 0.001; *χ*^2^_Weak_ = 74.43, *p* < 0.001), and cognitive impairment (*χ*^2^_Constrained_ = 6.42, *p* < 0.001; *χ*^2^_Weak_ = 22.61, *p* < 0.001). IPHRMs with a constrained capability set experienced statistically significantly lower levels of the following variables compared to the weak capability set: mental distance (*χ*^2^ = 13.25, *p* < 0.001) and emotional impairment (*χ*^2^ = 19.13, *p* < 0.001). Finally, IPHRMs with a robust capability set experienced statistically significantly lower levels of cognitive impairment than the constrained (*χ*^2^ = 6.42, *p* < 0.011) and weak (*χ*^2^ = 22.61, *p* < 0.001) capability sets. Also, IPHRMs with a robust capability set experienced statistically significantly lower levels of emotional impairment than the weak capability set (*χ*^2^ = 45.37, *p* < 0.001).

Hypothesis 1 is partially accepted, but in a different direction than expected. Cognitive demands were significantly higher in the robust capability set compared to the weak capability set. Hypothesis 2 is accepted. Job resources are associated with IPHRM practitioners’ capability sets. Furthermore, Hypotheses 3a to 3d are accepted: work capability sets of IPHRM practitioners are associated with their levels of exhaustion, mental distance, cognitive impairment, and emotional impairment.

## 5 Discussion

This study investigated the relationships between job demands, resources, work capability sets, and burnout among IPHRM practitioners. The study found three distinct capability sets among IPHRM practitioners (Van der Klink et al., 2016). The robust capability set comprised participants who had a high probability of achieving six of the seven capabilities, which may reflect a work context that enables individuals to convert resources and opportunities into meaningful functionings. The constrained capability set reflected a more limited freedom to achieve valued functionings. Participants in this set demonstrated two strong capabilities: using knowledge and skills, and developing new knowledge and skills. In contrast, the remaining capabilities, particularly earning a good income and being involved in important decisions, remained unrealized. These practitioners may be operating in undervalued roles (Uthman, 2024). The weak capability set represented individuals with minimal capability achievements across all dimensions. These findings reinforce the argument of the CA that merely providing resources is insufficient: what matters is whether individuals are free and enabled to achieve what they have reason to value in their professional lives (Robeyns and Byskov, 2025; Van der Klink et al., 2016). Therefore, addressing these disparities requires interventions that not only focus on resource allocation, but also aim to enhance conversion factors and eliminate systemic barriers to the full development of capabilities.

Practitioners in the robust group experienced higher cognitive demands than those in the weak group. This suggests that practitioners with robust work capability sets are equipped to engage with complex cognitive tasks and convert cognitive challenges into meaningful work outcomes (Van der Klink et al., 2016). By emphasizing agency, availability of resources, and individual context, the CA provides conceptual depth, suggesting that whether a demand is experienced as a strain or a challenge does not only depend on its objective nature, but also on the individual’s capability set and whether he/she is enabled to achieve meaningful outcomes. When demands are high, they are perceived as challenges rather than threats, inviting growth, not burnout (Kotze, 2018; Schaufeli et al., 2019). From the JD-R framework, cognitively demanding work can be a challenging aspect of work associated with engagement and professional growth when adequate resources are available. Similarly, the CA emphasizes that demanding work may remain compatible with wellbeing when individuals have the opportunity to translate their competencies into valued work outcomes.

The constrained capability set represents a conceptually rich configuration. IPHRM practitioners within this set reported high capabilities related to using and developing knowledge and skills and contributing to meaningful work, indicating strong professional competence and commitment. At the same time, capabilities related to participation in decision-making and earning a good income were markedly lower. This configuration reflects a situation in which individuals possess substantial professional resources and motivation but experience limitations in the institutional opportunities available to them (Sen, 2001). From a capability perspective, such patterns illustrate the distinction between personal competencies and the opportunity structures required to realize valued work outcomes.

IPHRM practitioners with robust and constrained capability sets reported significantly higher levels of co-worker support, role clarity, and autonomy compared to those with a weak capability set. Practitioners with a robust capability set also experienced significantly greater career progress than their counterparts with constrained and weak capabilities. These findings are consistent with previous research, which identifies job resources as central to capability development (Van der Klink et al., 2016) and, in particular, with their role in enabling meaningful goal setting, contribution, and autonomy (Nussbaum, 2000; Sen, 2005). Importantly, those with a robust capability set reported significantly lower levels of exhaustion, mental distance, cognitive impairment, and emotional impairment. IPHRMs with a robust capability set experienced lower levels of cognitive impairment than those with constrained and weak capability sets. While individuals with a constrained capability set exhibited lower mental distance and emotional impairment than those with a weak capability set, the robust group consistently demonstrated the most favorable outcomes across all wellbeing and career-related indicators.

This study confirms that job resources, such as role clarity, autonomy, career progress, and co-worker relationships, are associated with work capabilities, such as using and developing individuals’ knowledge and skills, taking part in key decisions, setting their own goals, developing and maintaining meaningful relationships, and contributing to something valuable (Barnard et al., 2023; Ragadu and Rothmann, 2025). Having job resources allows people to convert their potential into capabilities. The findings revealed meaningful distinctions among IPHRM practitioners with different capability sets in terms of both job demands and resources, as well as their experiences with burnout. Individuals with constrained and weak capability sets reported progressively diminished access to key job resources. This lack of support and clarity may inhibit their ability to realize valued capabilities, especially in areas such as autonomy and career development. Practitioners with a weak capability set appear to be structurally disadvantaged, indicating systemic barriers that are negatively associated with the expansion of their capabilities and limit their agency in the workplace.

The finding that the constrained capability set showed lower endorsement of earning a good income than the weak set suggests that earning a good income functions differently from other work-related capabilities. While the constrained group reported high opportunities across six domains, structural or institutional factors appeared to block the conversion of these capabilities into earning a good income. In Sen’s terms, this points to social, institutional, or market-based conversion factors that limit the translation of capability into functioning (Sen, 2001). It also highlights a form of “unfreedom” in which individuals are capability-rich yet economically constrained. This pattern resonates with Nussbaum’s (2000) observation that systemic arrangements may selectively undermine some central capabilities. The finding is also consistent with scholarship on the systematic undervaluation of feminized and care-related labor, where socially valuable work is nevertheless poorly remunerated (Budig and Misra, 2010; England, 2005). By contrast, the weak set may reflect adaptive preferences: individuals with consistently low endorsements may lower expectations across all domains, including income (Khader, 2011). Taken together, these results emphasize that capability sets are not additive; a stronger set on most dimensions does not guarantee broader flourishing. Instead, income appears structurally decoupled from other work capabilities, underscoring the need to analyze capability sets multidimensionally rather than assuming uniform enhancement across domains.

Role clarity outlines employees’ areas of influence and helps them become more aware of their goals. Without role clarity, IPHRM practitioners may feel disempowered (De Villiers and Stander, 2011), which negatively affects their capabilities to use their knowledge and skills with confidence, develop new knowledge and skills more purposefully, be involved in important decisions, set their own goals, and work together with others without friction. Role clarity aligns tasks with purpose, autonomy supports control, and good co-worker relations reinforce impact, which supports the capability to contribute to something valuable (Nussbaum, 2011; Sen, 2005).

Job autonomy provides individuals with the space and encouragement to engage in self-directed learning and experimentation, thereby enhancing their ability to integrate external influences into their values (Bradshaw and Ryan, 2026). Also, autonomy provides individuals with opportunities to shape their targets and act on their skills or interests, thereby affecting their ability to develop new knowledge and skills, be involved in important decisions, and set goals (Duong and Pham, 2023). Career progression offers training pathways that allow individuals to develop new knowledge and skills. Individuals who lack career progress may not feel capable of defining the knowledge and skills they should use and develop, which may result in burnout and put the sustainability of their careers at stake (De Vos et al., 2020; De Vos and Van Der Heijden, 2017). Good co-worker relationships, which are regarded as a vital job resource, foster trust and a sense of belonging and promote social learning, thereby enhancing work capabilities and reducing burnout (Barnard et al., 2023; Day and Leiter, 2018).

In this study, burnout patterns closely mirrored the capability sets. Those with a robust capability set reported lower levels of exhaustion, detachment, and cognitive and emotional impairment, suggesting that enabling conditions in the work environment not only support capability realization but also help prevent burnout. Individuals in the constrained and weak groups experienced significantly greater burnout, with the weak group most acutely affected (Gürbüz et al., 2024). These patterns underscore the vital role of capability-enhancing work environments in fostering conditions that not only provide resources, but are also associated with individuals’ genuine freedom to achieve what they have reason to value in their professional lives (Bradshaw and Ryan, 2026).

The findings of this study are aligned with the JD-R model and the CA. Weak capabilities imply that individuals lack the opportunity, support, or conditions that they need to achieve valued beings and doings (Van der Klink et al., 2016). When IPHRM practitioners value certain beings or doings (e.g., developing new knowledge and skills, being involved in decision-making, and earning a good income), but cannot pursue them due to organizational constraints that limit their freedom, frustration arises, which leads to exhaustion (Duong and Pham, 2023). Moreover, suppose that IPHRM practitioners cannot choose and act on what is important to them. In that case, they will experience alienation, which results in mental distance towards their work and others (Bradshaw and Ryan, 2026). Furthermore, IPHRM practitioners who struggle to convert resources into capabilities may develop burnout.

The results of this study suggest that expanding the conditions (e.g., role clarity, autonomy, and supportive relationships) that enable capability conversion is central for promoting social justice and sustainable employability in this profession. IPHRM practitioners with robust capability sets reported higher co-worker support, clearer roles, and greater autonomy, suggesting more favorable conversion conditions. Those with a weak capability set had access to fewer real choices, which points to underlying structural or cultural barriers. The burnout patterns support this view: robust capability sets were associated with lower levels of exhaustion, mental distance, cognitive impairment, and emotional impairment. From a capability perspective, these differences signal unequal opportunities to maintain wellbeing, which raises concerns about social justice in everyday work. These differences also matter for sustainable employability (Abma et al., 2016; Van der Klink et al., 2016). When capability sets are constrained or weak, both vitality and growth are limited, which restricts meaningful participation over time.

The study’s contribution lies less in the occupational group examined and more in the conceptual integration of the JD-R framework with the CA, which provides a richer account of how work conditions relate to employees’ opportunities to achieve valued work functionings. Adopting a capability perspective changes the interpretation of work conditions in several important ways relative to traditional JD-R studies (der Van Klink, 2019). Whereas the JD-R model focuses primarily on how job demands and resources relate to wellbeing outcomes such as engagement or burnout (Bakker et al., 2014), the capability approach emphasizes individuals’ real opportunities to achieve valued work functionings (Van der Klink et al., 2016). From this perspective, the presence of job resources alone is not sufficient; what matters is whether employees are able to convert these resources into meaningful opportunities to use their skills, participate in decisions, and contribute to valued goals (Abma et al., 2016; der Van Klink, 2019). This shift in focus highlights how similar work conditions may lead to different capability configurations across individuals, reflecting variations in opportunity structures and conversion factors.

An important implication of the findings concerns the potential role of organizational practices in shaping capability configurations. Capability sets reflect the opportunities individuals perceive to achieve valued work functionings. These opportunities are partly embedded in organizational arrangements, such as role clarity, participation in decision-making, access to supportive relationships, and fair reward structures. Interventions that strengthen these opportunity structures may therefore be associated with more favorable capability configurations among practitioners. However, given the cross-sectional nature of the present study, these interpretations should be regarded as theoretically informed implications rather than evidence of causal effects.

## 6 Limitations and recommendations

This study had various limitations. The cross-sectional and correlational nature of the study limits the extent to which causal interpretations can be made from the observed relationships (Spector, 2019). Accordingly, the theoretical implications reflect conceptual interpretations informed by the CA and JD-R model rather than direct empirical tests of causal mechanisms. Future research could also benefit from adopting longitudinal or mixed-method designs to trace the dynamic interplay between contextual work factors, leadership practices, and practitioners’ capability development, thereby offering a richer understanding of how sustainable employability unfolds in practice (Vivek and Nanthagopan, 2021). Furthermore, the non-probability sample restricts the representativeness of the findings (Cruz, 2021).

A further limitation concerns the reliance on self-reported perceptions of work conditions and capabilities (Spector, 2019). Although the study discusses structural inequality and justice-related issues, the data capture how practitioners experience their work environment rather than objective organizational indicators such as contract arrangements and workload metrics. From a capability perspective, individuals’ perceptions of available opportunities and constraints are conceptually relevant because they reflect how work conditions are experienced and interpreted in practice. However, the absence of objective indicators limits the ability to assess structural conditions within organizations. Future research would benefit from combining subjective capability measures with organizational-level indicators to provide a more comprehensive assessment of structural inequality in professional work contexts.

The use of professional networks for recruitment may have introduced self-selection bias, as participation was voluntary and may have attracted practitioners who were more professionally engaged or interested in wellbeing-related topics. Consequently, practitioners experiencing higher levels of strain or marginalization may be underrepresented. While this approach facilitated access to a relevant professional sample, caution is warranted when generalizing the findings.

Dichotomizing aspects of capabilities in this study is a limitation (Fedorov et al., 2009). Dichotomizing continuous variables is generally discouraged because it entails a loss of information and statistical power. However, there are circumstances in which categorization can be theoretically justified. In the present study, dichotomization was not applied to simplify analyses or to create artificial group comparisons, but to consolidate multiple dimensions of each capability aspect into a single capability indicator. This approach aligns with the conceptual logic of the CA, which focuses on whether individuals are effectively able to achieve valued functionings, rather than on fine-grained variation in underlying indicators (Abma et al., 2016).

Another limitation of the study relates to analytical and statistical issues. The relatively small sample size constrained the analysis of demographic differences. Demographic variations in job demands and resources, work capabilities, and burnout should be investigated in future studies. Furthermore, the available sample size precluded a split-sample validation strategy (Kline, 2023). Instead, a single-sample approach combining confirmatory factor analyses and reliability estimation was employed. This approach provided preliminary support for the scales’ validity in the present context; however, further validation is required with larger samples. Additionally, the identification of capability sets was sample-specific, and the results of latent class analyses may vary across samples (Spurk et al., 2020). Replication with larger, more representative samples is necessary.

The findings of this study have practical implications for how organizations might support robust capability sets among IPHRM practitioners. Capability-oriented interventions can operate at multiple levels of the work environment (Meerman et al., 2022). At the individual level, structured job-crafting initiatives may enable employees to shape aspects of their work to better align with their skills and professional goals, thereby increasing opportunities to use and develop their knowledge and expertise (Baka et al., 2025). At the team level, participatory decision-making practices and inclusive leadership behaviours may enhance practitioners’ involvement in important organizational decisions and support the development of meaningful relationships (Randel et al., 2018; Shore and Chung, 2022). At the organizational level, policies that promote role clarity, fair reward structures, and access to development opportunities may strengthen the opportunity structures through which practitioners experience and realize valued work outcomes (Jo and Shin, 2025).

## 7 Conclusion

The findings highlight the significant role of robust capability sets against burnout among IPHRM practitioners. Those who accessed meaningful opportunities (autonomy, role clarity, and co-worker support) were less likely to experience exhaustion, mental distance, and psychological impairment. In contrast, constrained and weaker capabilities were strongly associated with increased burnout symptoms. Job demands and resources were consistently associated with differences in practitioners’ capability sets and wellbeing outcomes. These conditions are positioned as contextual features rather than proximal outcomes, reflecting their role as background structures within which practitioners experience their work. This positioning aligns with the CA, which conceptualizes work conditions as part of the opportunity structure that shapes individuals’ freedom to achieve valued functionings.

A central contribution of the model is the placement of work capabilities as an intermediate layer linking work conditions to indicators of functioning (burnout). Rather than treating wellbeing outcomes in isolation, the findings suggest that variations in functioning were consistently patterned by differences in capability sets. This supports treating capabilities as an integrative construct that captures how work conditions are experienced as meaningful opportunities, extending beyond traditional attitudinal frameworks. By integrating CA and burnout literature, this study highlights structural inequities within professions and illustrates how enabling or constraining environments influence practitioners’ functioning and wellbeing. This study emphasizes the importance of designing work environments that foster capability expansion, not only to improve performance, but also to safeguard psychological health and affirm human dignity in the workplace.

## Data Availability

The datasets presented in this study can be found in online repositories. The names of the repository/repositories and accession number(s) can be found below: Rothmann, Sebastiaan (2025), “IPHRM Demands, Resources, Capabilities and Burnout,” Mendeley Data, V1, doi: 10.17632/rysk5fbnsk.1.
